# Cooperative Spatial Retreat for Resilient Drone Networks †

**DOI:** 10.3390/s17051018

**Published:** 2017-05-03

**Authors:** Jin-Hyeok Kang, Young-Min Kwon, Kyung-Joon Park

**Affiliations:** Department of Information and Communication Engineering, Daegu Gyeongbuk Institute of Science and Technology (DGIST), Daegu 42988, Korea; kang@dgist.ac.kr (J.-H.K.); kym9102@dgist.ac.kr (Y.-M.K.)

**Keywords:** drones, unmanned aerial vehicles, aerial networks, spatial retreat

## Abstract

Drones are broadening their scope to various applications such as networking, package delivery, agriculture, rescue, and many more. For proper operation of drones, reliable communication should be guaranteed because drones are remotely controlled. When drones experience communication failure due to bad channel condition, interference, or jamming in a certain area, one existing solution is to exploit mobility or so-called spatial retreat to evacuate them from the communication failure area. However, the conventional spatial retreat scheme moves drones in random directions, which results in inefficient movement with significant evacuation time and waste of battery lifetime. In this paper, we propose a novel spatial retreat technique that takes advantage of cooperation between drones for resilient networking, which is called cooperative spatial retreat (CSR). Our performance evaluation shows that the proposed CSR significantly outperforms existing schemes.

## 1. Introduction

Networking has become an essential element of daily life, especially because of rapid advances in the Internet of things (IoT) [1] and smartphones. Thus, people and devices are perpetually connected to networks and rely on them for routine functions. Consequently, it is critically important to provide a reliable network infrastructure for various kinds of applications in cyber-physical systems [2,3]. However, emergency networking, such as disaster areas, requires extensive time for service providers to deploy additional infrastructure [4]. If network service is sufficient in disaster areas, people can notify their families about their safety or and location in case they become isolated in a collapsed structure. Even if a person is unconscious, adequate network service can enable venders to find his or her approximate location via localization schemes. Currently, network service venders temporarily extend networks by using communication relay vehicles. This is a common and sufficient solution when roads are clear and intact. However, in certain disasters such as earthquakes, tsunamis, and typhoons, we cannot realistically expect the roads to be clear enough for communication relay vehicles to move into disaster areas.

**Figure 1:**
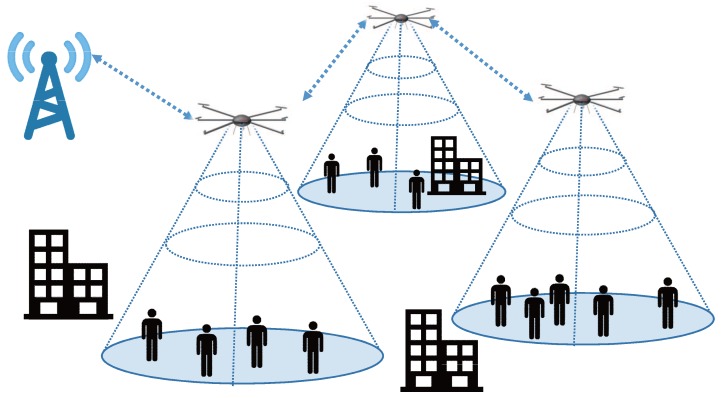
Illustration of net-drones.

Therefore, we can use drones for a significantly reducing deployment time of network infrastructure. More specifically, aerial networks or so-called *net-drones* [5] illustrated in Figure 1 can be used to reconstruct network infrastructure without deploying underground infrastructure. The most important advantage of net-drones is their uninhibited mobility. Conventional vehicles can only move in two dimensions on the ground while net-drones exploit mobility in the three-dimensional space, which enables drones to maneuver over obstacles. Thus, drones may be a promising replacement for emergency response vehicles [6]. However, to this end, we need to resolve certain technical challenges. In particular, it is of critical importance how to provide reliable communication under bad channel condition, interference, and even jamming.

In this paper, we focus on resilient wireless networking of net-drones over communication failures due to bad channel condition, interference, or jamming in certain areas. There are mainly two directions to avoid communication failures, i.e., channel surfing [7] and spatial retreat [8]. Since channel surfing is a partial solution, especially under severely deteriorated channel conditions, we consider spatial retreat, which exploits the mobility of drones, as a more comprehensive solution for resolving communication failures in certain areas. In particular, we propose an efficient spatial retreat mechanism, cooperative spatial retreat (CSR), which evacuates drones from the communication failure areas in a cooperative manner. Unlike the conventional spatial retreat scheme that moves drones in random directions [8], the proposed CSR scheme collects location information from drones in the communication failure area by exploiting telemetry modules. Then, by estimating the central point of communication failure based on the location information, CSR efficiently evacuate drones from the failure area.

The remainder of this paper is organized as follows. In Section 2, we provide background net-drones. In Section 3, we describe the proposed spatial retreat algorithm of CSR. Then, in Section 4, we describe the performance of the CSR algorithm and compare the performance evaluation results to those using the conventional techniques. Finally, we give our conclusions in Section 5.

## 2. Background

First, we introduce the concept of net-drones. Then, we explain localization methods. In a nutshell, the proposed scheme, called cooperative spatial retreat (CSR), collects location information from nearby drones via telemetry modules in the communication failure area. Then, based on the information, it estimates the failure region and evacuate drones in the opposite direction of the center of the failure region to reduce the evacuation time. To this end, here we summarize localization techniques and give the details of the CSR algorithm in the next section.

### 2.1. Net-Drones

In the first half of the 20th century, drones were developed for military purposes, specifically to perform scouting, monitoring, and bombing missions. Recently, many global companies have invested in R & D for commercial applications of drone technology. Already, drones used for broadcasting are very common, and most broadcasting companies operate drones for movies, narrative television, and sports matches. Another use of drones is providing network infrastructure. In fact, Google and Facebook are trying provide Wi-Fi service for developing countries via drones.

The net-drone is proposed as a mobile ad hoc networks consisting of drones for emergency network service [5]. When disasters occur, significant parts of the network service infrastructure could be destroyed. In addition, consumption of network bandwidth rapidly increases in disaster areas due to abruptly increasing use of the service. Normally, network service venders temporarily expand bandwidth via relay vehicles, but these vehicles often cannot reach disaster areas. In this case, net-drones could replace relay vehicles and enhance emergency responses. In the case of Titan and Aquila, a single drone covers a large area, while net-drones cover areas via a drone fleet. Thus, net-drones require reliable communication connectivity to provide network service.

### 2.2. Localization Techniques

The proposed CSR technique requires location information of drones. Hence, the reliability of localization is important for our scheme. GPS is a common technique to find a given location; however, it has several limitations including its reliability. In non-line-of-sight (NLOS) situations such as indoor environments, GPS is unable to find specific locations [9], which can be a serious issue for net-drones because controllers may lose command over drones. Here, we present a brief survey of effective solutions for complementing GPS for net-drones. We categorize localization techniques to find a suitable option for net-drones.

**Figure 2:**
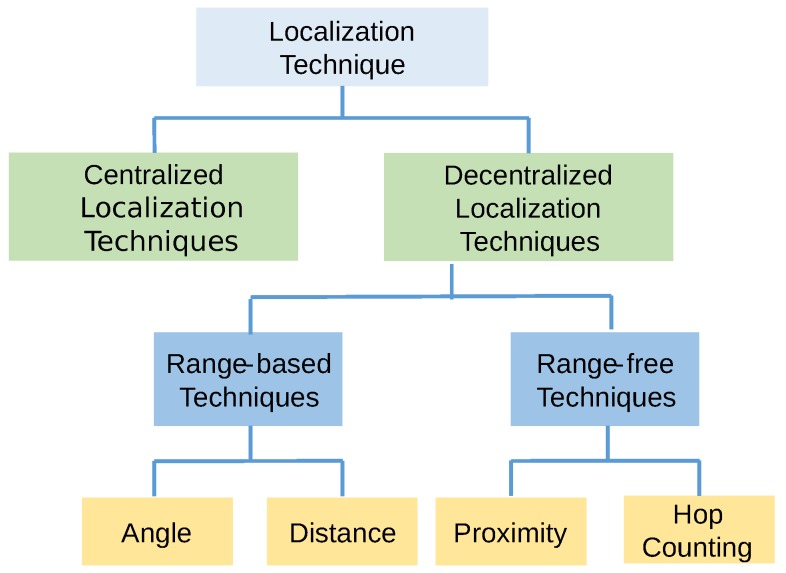
Sub-categories of localization techniques.

According to [10], localization techniques can be divided into two categories, i.e., centralized and decentralized, by the nodal organization structure. Decentralized localization is further divided into two categories based on how to calculate locations between each node. These comprise range-based techniques and range-free techniques, as shown in Figure 2 [11]. Many localization techniques used in the field are range-based techniques, which can be further divided in terms of their characteristics, such as angle and distance [12]. Range-free methods can also be divided via local hop counting [13]. Similarly, localization techniques can be divided into anchor/beacon-based or anchor/beacon-free; GPS-based or GPS-free; fine-grained or coarse-grained; and stationary or mobile sensor nodes, among others [14].

#### 2.2.1. Centralized Localization Techniques

Centralized localization techniques transmit data to a central node, and the central node computes location information about each individual node. In [15], the authors propose a centralized technique, which is a method for estimating unknown node positions via convex optimization. This technique is based on connectivity-induced constraints. Conversely, MDS-MAP [16] is an algorithm that uses connectivity information through multidimensional scaling (MDS). MDS-MAP uses much less information and recovers more accurate maps of node location.

These centralized localization techniques take advantage of the fact that nodes do not need expensive and sophisticated sensors such as GPS. Another advantage is that nodes do not require computational costs to calculate locations because the central node is responsible for all computation. However, centralized localization techniques also have some disadvantages. For example, every node sends information to the central node, which requires significant communication cost, large bandwidth usage, and longer delay. In particular, the communication cost is a serious problem for net-drones because drones are usually very sensitive to battery consumption. Frequent communication to the central node requires higher energy consumption. Consequently, centralized localization techniques may be unsuitable for net-drones.

#### 2.2.2. Decentralized Localization Techniques

Decentralized localization techniques transmit data to nearby nodes. As such, they do not rely on centralized computation, so they are able to determine their locations with limited communication. Thus, this approach is more suitable for net-drones than centralized localization techniques. Decentralized techniques can be classified as range-based and range-free techniques.

##### Range-Based Localization Techniques

These techniques estimate the distance or angle between nodes, and find their location primarily by trilateration. These range-based techniques include most common localization techniques such as GPS, RSSI, TOA, TDOA, AOA, etc. Typically, more accurate range-based localization techniques are complex. Consequently, how to mitigating this tradeoff is a critical issue.

##### Range-Free Localization Techniques

Range-free localization is mainly classified into two categories, hop counting and local techniques. Importantly, the accuracy of the location estimate is usually smaller than that of range-based localization techniques. Local methods rely on a high nodal density. In particular, the centroid localization technique [17] is a range-free, proximity-based, coarse-grained localization technique that is suitable for small, energy efficient nodes without GPS. Functionally, it is based on the spherical radio propagation assumption. This technique finds a location by calculating the center of the locations of all nodes it can detect. Alternatively, the approximate point in triangulation (APIT) localization technique [18] utilizes an area-based, range-free localization approach. This technique requires a heterogeneous network of sensing devices to employ a novel, area-based approach to estimate location. On the other hand, hop counting methods rely on flooding. The distance vector-hop (DV-Hop) localization technique [19] uses a similar mechanism to that of classical distance vector routing to estimate the distance between unknown nodes and reference nodes, expressed as the product of the average hop distance and hop count [20].

## 3. Cooperative Spatial Retreat (CSR)

### 3.1. Underlying Idea of CSR

**Figure 3:**
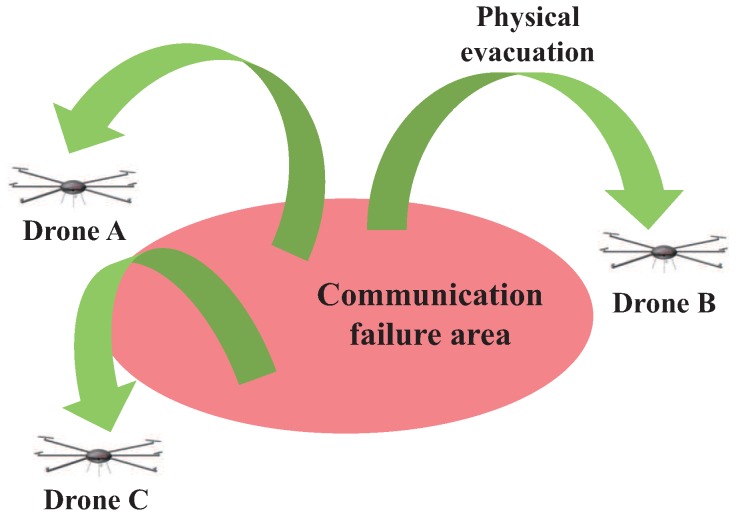
Concept of spatial retreat.

A communication-wise strategy in a communication failure area is channel surfing [7], which is similar to frequency hopping. A different method is to exploit the mobility of drones called spatial retreat [8], which physically moves drones outside the failure area. When drones experience degraded channel condition, the conventional spatial retreat scheme enables drones to escape from the communication failure area in a random manner, as shown in Figure 3. However, random escape is inefficient and may waste energy and deplete batteries.

**Figure 4:**
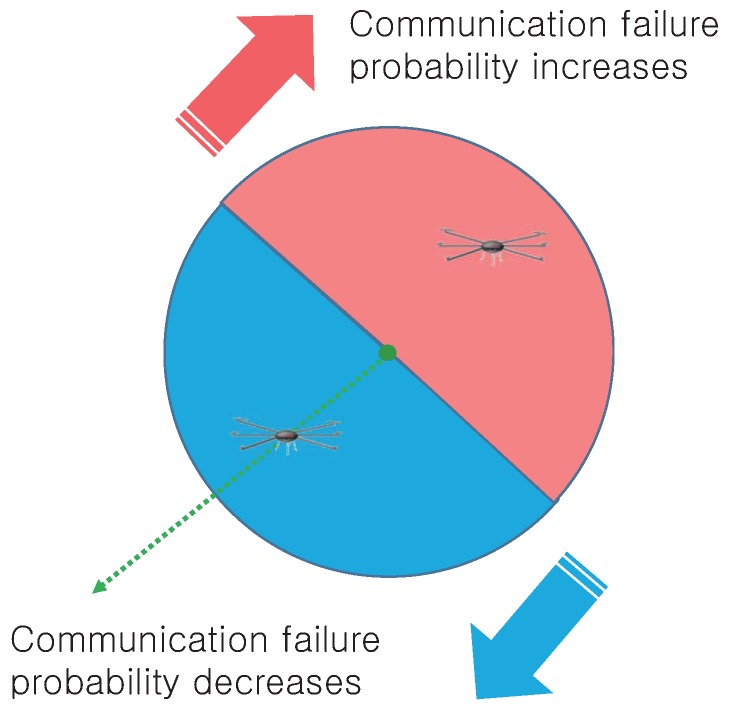
Idea of cooperative spatial retreat.

Hence, we propose a mechanism called cooperative spatial retreat (CSR) for improving the efficiency of the conventional spatial retreat. The key difference between CSR and the conventional spatial retreat is that CSR further exploits information obtained from additional communication via assistance telemetry modules. In the conventional spatial retreat scheme, drones choose the evacuation direction in a random manner. Conversely, the proposed scheme exploits location information from other drones to prompt a drone to move in a particular direction with a high probability of improving the channel condition. The main function of CSR is to collect the location information of nearby drones in the communication failure area and to estimate the failure region so that drones can evacuate in the opposite direction of the area as shown in Figure 4.

### 3.2. Cooperative Spatial Retreat Algorithm

The proposed cooperative spatial retreat (CSR), detailed in Algorithm 1, involves an algorithm that utilizes a target drone and its neighboring drones (cooperative drones) to send location information to the target drone. First, at the DETECT_FAILURE sequence, the target drone needs to detect whether or not it is in the communication failure area by considering information such as the received signal strength indicator (RSSI), packet delivery ratio, carrier sense time, etc. [21]. Importantly, when drones are moving to mission areas, evacuation movement might not be required. However, doing so makes it probable that drones will be passing communication failure areas. Here, drones wait before arriving at their destination by following the DRONE_MOVING sequence. However, if drones are still suffering from communication failure upon arrival, they continue to loop through the CSR algorithm.
**Algorithm 1** Cooperative spatial retreat of net-drones 1:**procedure** Cooperative spatial retreat 2:  **if** DETECT_FAILURE=true **then** 3:    **if** DRONE_MOVING=true **then** 4:      move_toward_mission_area() 5:    **else** 6:      turn_on_telemetry() 7:      **if** inner_drones<=2 **then** 8:        **for** each_drone(i)<number_of_drone **do** 9:          get_outside_drone_location()10:          **if** channel_error=true **then**11:            **go to** 912:          **end if**13:        **end for**14:      **else**15:        **for** each_drone(i)<number_of_drone **do**16:          get_inside_drone_location()17:          **if** channel_error=true **then**18:            **go to** 1619:          **end if**20:        **end for**21:      **end if**22:      set_midpoint()23:      evacuate()24:      reconstruct_phase()25:    **end if**26:  **else**27:    normal_phase()28:  **end if**29:
**end procedure**



In this scheme, each drone that stays in the communication failure area acts as a target drone. The target drone communicates with neighbor drones in the communication failure area by using the assistance communication module of telemetry. This action is triggered by a “turn_on_telemetry” sequence. At this point, target drones collect location information from other drones in the communication failure area [22]. It should be noted that telemetry modules using a different frequency band from main communication are already available in drones, which may be unsuitable for real-time two-way data communication, but sufficient for transmitting the one-way location information that is an extremely low rate. Consequently, location information collected from cooperative drones can be used to calculate directions to evacuate from the communication failure area.

However, if there are no cooperative drones in the communication failure area, target drone cannot collect the location information from others. Therefore, it is necessary to classify whether or not the cooperative drones exist in the communication failure area. So, first identify the number of drones in the communication failure area and choose whether to cooperate internal drones or external ones.

**Figure 5:**
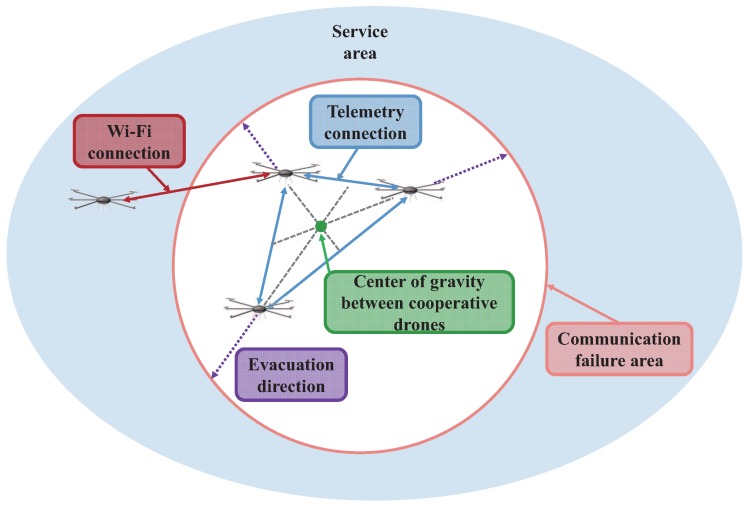
Mechanism of cooperative spatial retreat.

When the target drones collect the location information from the cooperative drones, the location data may not be transmitted due to bad channel condition. This situation is executed at the “channel_error()” sequence. In this case, drones retransmit the location information until target drones collect all of the location data. This action is executed at the “get_outside_drone_location” or “get_inside_drone_location” sequence. Consequently, the target drone can calculate the center of gravity between cooperative drones and itself using the “set_midpoint()” sequence. With this information, the target drone can run an evacuate sequence in the exact opposite direction from the center, as shown in Figure 5. In addition, the drone fleet also needs to run a reconstruction [23] phase to reorganize its position data after running the CSR algorithm for optimal communication.

### 3.3. Remarks on the Performance of the CSR Scheme

**Figure 6:**
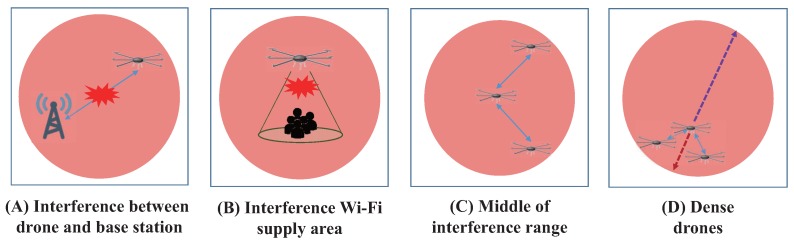
Limitations of cooperative spatial retreat.

There are some situations when the proposed CSR algorithm may not properly work, as shown in Figure 6. For example, Situation (A) depicts interference occurring at the base station. Even though net-drones comprise an ad-hoc network, they still need to connect with a base station to provide Internet service for users. Moreover, Situation (B) depicts Internet service users suffering from interference. Situation (C) shows a target drone in the middle of an interference area, where the CSR becomes a random escape instruction sequence. In this case, the CSR algorithm provides the same moving distance as a random evacuation. Finally, Situation (D) shows that drones are mostly located near the boundary of the failure area. In this case, the estimated center of the failure area will be inaccurate, and the performance of the CSR technique may degrade. We call this issue the dense boundary problem.

## 4. Performance Evaluation

In this section, we compare the performance of the CSR with conventional schemes. In particular, we compare the moving distance for evacuation from the communication failure area. The parametric values used in our simulation study are summarized in Table 1.

### 4.1. Comparison of Schemes

**Figure 7:**
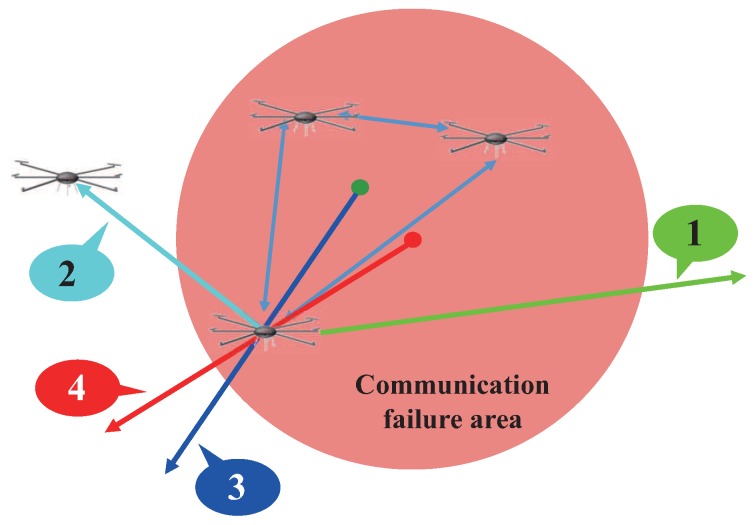
Illustration of drone movement by each of four schemes.

**Figure 8:**
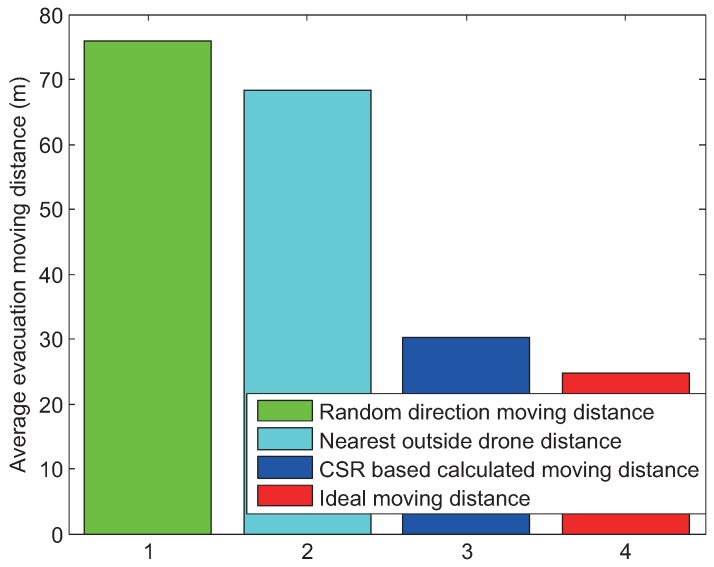
Evacuation distance for each scheme.

In our simulation study, we compare four algorithms including the proposed CSR scheme. Figure 7 illustrates each scheme while Figure 8 shows how far the target drone moves to evacuate the communication failure area. The first scheme denoted by 1 in Figure 7 is the conventional spatial retreat scheme that randomly evacuate the drone from the communication failure area. Hence, it gives the worst performance on average.

The second method denoted by 2 is one that evacuates the target drone to the nearest drone outside of the communication failure area. The third algorithm denoted by 3 is the proposed cooperative spatial retreat (CSR) technique; whereas, the last algorithm denoted by 4 is the ideal case, where the target drone knows perfect information on the failure area and moves out in the exactly opposite direction of the area.

As given in Figure 8, the average moving distance of the CSR is less than half of that of the conventional scheme. Furthermore, the performance of the CSR is comparable to that of the ideal scheme with full knowledge of the communication failure area. In the following section, we will further evaluate the performance of the schemes.

### 4.2. Influence of the Size of the Communication Failure Area

**Figure 9:**
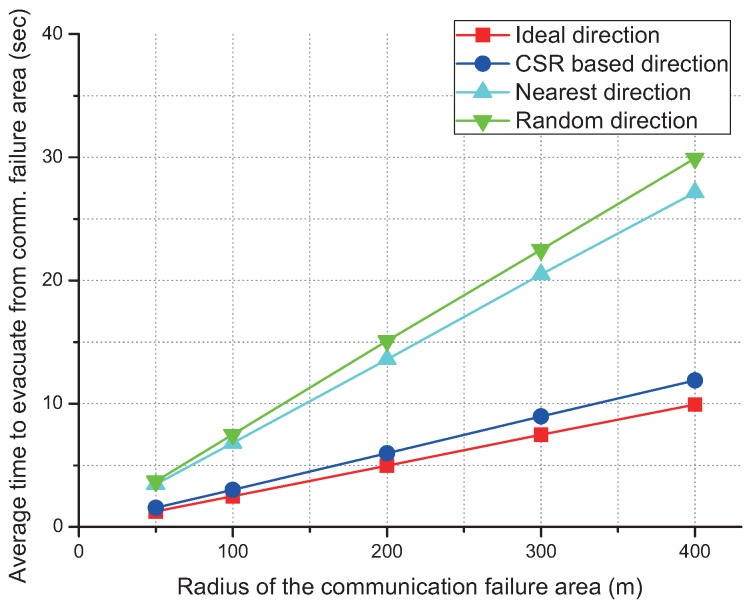
Average time to evacuate for different values of the radius of the communication failure area.

First, we conduct simulation on the effect of the communication failure area size as shown in Figure 9. Each simulation result uses 3 inner drones and 3 outside drones. Also, simulation is an average value over 10,000 times per each graph. Other parameters are the same as in Table 1. There are five simulation results regarding the influence of the communication failure area size. The evacuation distance increases as the size of the communication failure area increases. However, the relative performance between the schemes changes little. Hence, we can conclude that the size of the communication failure area does not much affect the relative performance of the schemes.

### 4.3. Evacuate to the Nearest Outside Drone

**Figure 10:**
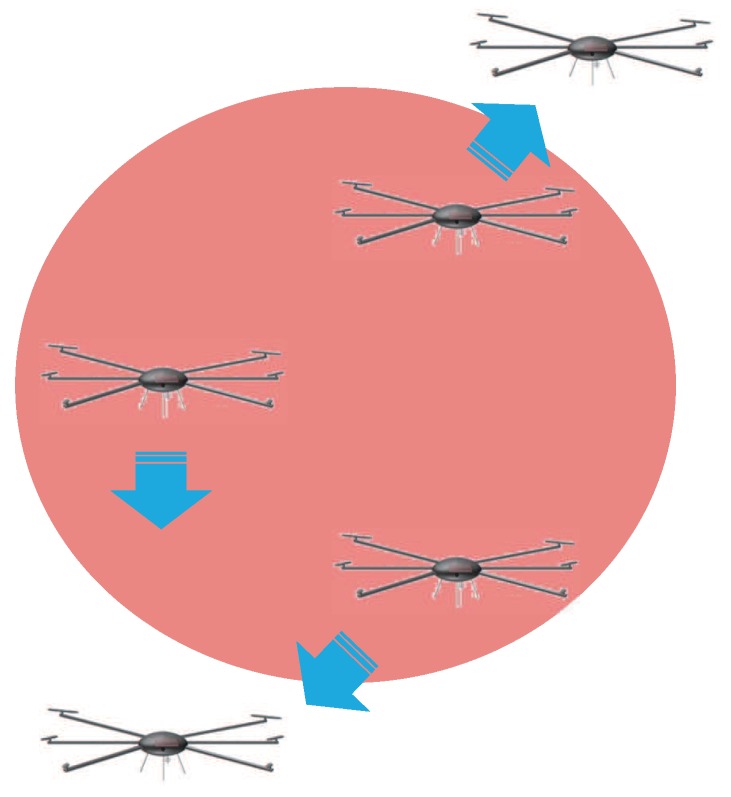
Idea of evacuation to the nearest drones.

**Figure 11:**
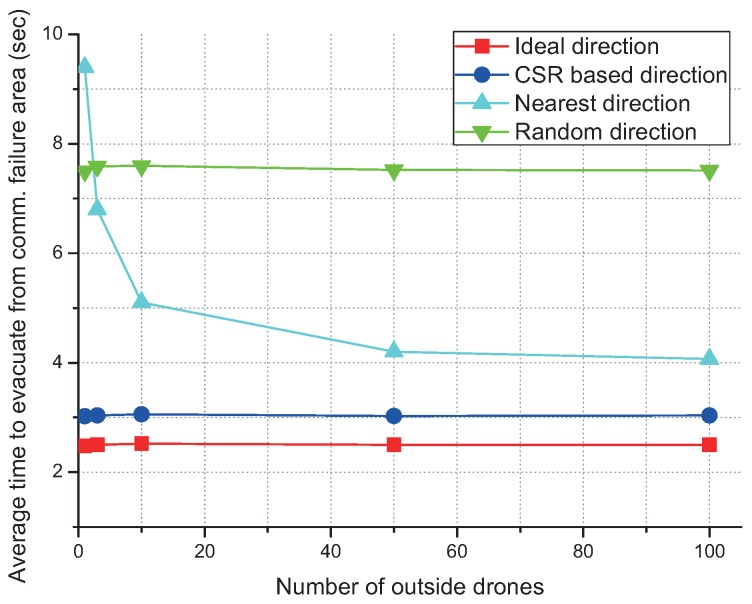
Average time to evacuation according to the different number of outside drones.

Possibly, one of the simplest way to avoid the failure area is to move to the nearest outside drone as shown in Figure 10. However, the efficiency of this idea is not as good as the CSR algorithm. Efficiency of the evacuation distance increases as the number of outside drones increases. However, even with hundreds of outside drones, the average time to evacuation is comparable with that of the random evacuation as shown in Figure 11.

### 4.4. Cooperative Spatial Retreat

**Figure 12:**
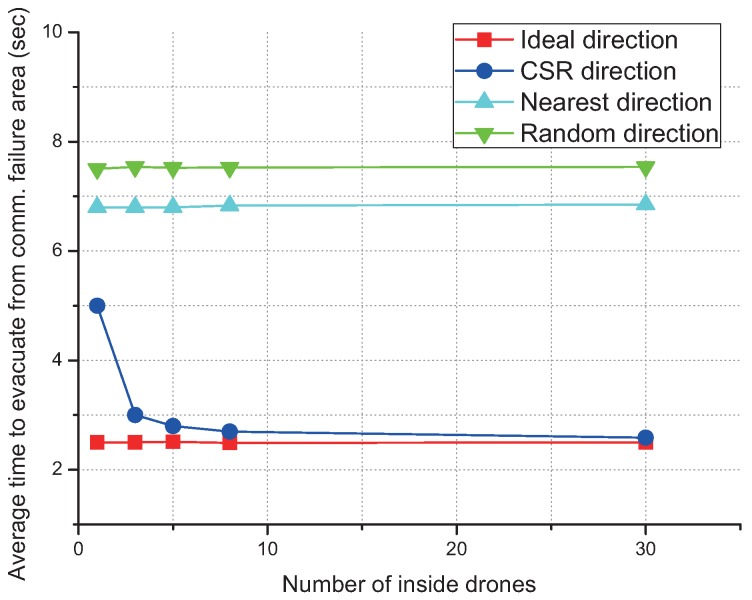
Average time to evacuation according to the different number of inside drones.

As the number of cooperative drones increases, CSR rapidly outperforms random evacuation and becomes similar to the ideal scheme as shown in Figure 12, which shows that the CSR gives near-optimal performance under cooperation with as many as only three drones.

## 5. Conclusions

In this paper, we have proposed an efficient spatial retreat scheme, cooperative spatial retreat (CSR), to enhance the communication reliability of net-drones. Drones cooperatively share their communication channel information in CSR, which significantly enhances the retreat performance compared to the existing method. In addition, we have further considered three-dimensional evacuation while the conventional scheme only considers two-dimensional cases. Our performance evaluation shows that the proposed CSR scheme can significantly reduce the evacuation time as well as the battery consumption of drones by efficient movement.

## Figures and Tables

**Table 1:** Parameters used in the simulation study.

Parameter	Value
Simulation tool	MATLAB (R2013a 8.1.0.604)
Shape of the failure area	Circle
Radius of the failure area	100 m
Drone flying speed	10 m/s
Maximum evacuation distance	200 m
Channel error probability	0.01
